# A Common Stay-on-Goal Mechanism in the Anterior Cingulate Cortex for Information and Effort Choices

**DOI:** 10.1523/ENEURO.0454-24.2025

**Published:** 2025-03-04

**Authors:** Valeria V. González, Melissa Malvaez, Alex Yeghikian, Sydney Wissing, Melissa Sharpe, Kate M. Wassum, Alicia Izquierdo

**Affiliations:** ^1^Department of Psychology, University of California-Los Angeles, Los Angeles, California 90095; ^2^Department of Psychology, University of Sydney, Sydney, New South Wales 2006, Australia; ^3^The Brain Research Institute, University of California-Los Angeles, Los Angeles, California 90095; ^4^Integrative Center for Learning and Memory, University of California-Los Angeles, Los Angeles, California 90095; ^5^Integrative Center for Addictions, University of California-Los Angeles, Los Angeles, California 90095

**Keywords:** effort-based decision-making, frontal cortex, information choices, reward learning, uncertainty, value-based decisions

## Abstract

Humans and nonhumans alike often make choices to gain information, even when the information cannot be used to change the outcome. Prior research has shown that the anterior cingulate cortex (ACC) is important for evaluating options involving reward-predictive information. Here we studied the role of ACC in information choices using optical inhibition to evaluate the contribution of this region during specific epochs of decision-making. Rats could choose between an uninformative option followed by a cue that predicted reward 50% of the time versus a fully informative option that signaled outcomes with certainty but was rewarded only 20% of the time. Reward seeking during the informative S+ cue decreased following ACC inhibition, indicating a causal contribution of this region in supporting reward expectation to a cue signaling reward with certainty. Separately in a positive control experiment and in support of a known role for this region in sustaining high-effort behavior for preferred rewards, we observed reduced lever presses and lower breakpoints in effort choices following ACC inhibition. The lack of changes in reward latencies in both types of decisions indicate the motivational value of rewards remained intact, revealing instead a common role for ACC in maintaining persistence toward certain and valuable rewards.

## Graphical abstract

**Figure:**
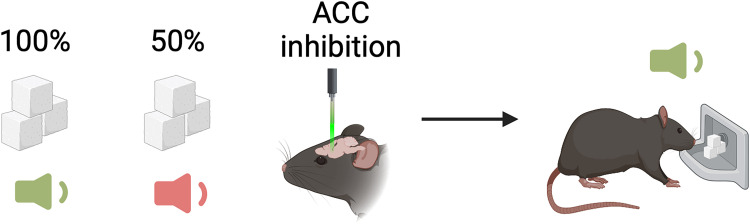
Visual Abstract

## Significance Statement

We often make choices to gain information, even when the information cannot be used to change the outcome. Here we investigated the precise timing of the role of the anterior cingulate cortex (ACC) in decisions that involve seeking certain versus uncertain rewards. By optically inhibiting ACC neurons, we demonstrate that this region is crucial for maintaining persistence toward rewards signaled with certainty, without altering the motivational value of the reward itself. In a positive control experiment, we also confirm that ACC is important in effort-based choice. The findings reveal a common role for ACC in maintaining persistence toward certain and valuable rewards, necessary for making optimal decisions. These results have implications for understanding psychiatric disorders involving maladaptive reward-seeking behavior.

## Introduction

Individuals often are willing to pay a cost for information even when the information cannot be used to change the outcome. Prior research has shown that information-seeking behavior is modulated by a network involving the frontocortical regions such as the anterior cingulate cortex (ACC) and orbitofrontal cortex, along with subcortical regions like the striatum and pallidum (Bromberg-Martin and Monosov, 2020). Indeed, macaque ACC activity is one the earliest predictors of future choices suggesting it is important in establishing the drive to actively seek information from the environment (White et al., 2019). By comparison, research on the specific brain regions tracking information value in rodents is scarce (Bussell et al., 2023), as are inquiries about the causal mechanisms involved. Recently, we found that ACC was critical in information choice preference and that its mechanism was sex dependent: previously rewarded information choices strongly predicted future choices in control animals, but not in female rats following ACC inhibition. These results revealed a causal role for ACC in decisions involving information (González et al., 2024).

To model information choices in nonhumans, several groups (Stagner and Zentall, 2010; Molet et al., 2012; Pisklak et al., 2015; Trujano and Orduña, 2015; Vasconcelos et al., 2015; Macias et al., 2021) administer a task where rats choose between a more profitable option followed by a discrete cue, rewarded 50% of the time (No information) versus a less profitable option followed by a cue that signals a certain outcome (Information). One of these cues (S+) always results in reward (100%) and the other (S−) is never rewarded (0%). In this procedure, the information follows the choice and does not affect the outcome and thus, any preference for that option suggests information is inherently rewarding (Stagner and Zentall, 2010; Trujano and Orduña, 2015; Cunningham and Shahan, 2019; Macias et al., 2021). A preference for information could be due to an overweighting of the cue that signals certain reinforcement (S+) and/or an underweighting of the cue paired with the absence of reward (S−) (McDevitt et al., 2016; González and Blaisdell, 2021; González et al., 2023). However, behavioral manipulations involving either the S+ or S− have led to largely equivocal results, with more studies garnering support for overweighting the S+ (Stagner et al., 2011; Fortes et al., 2016).

To more precisely uncover the timing of the role of ACC in information choices, we studied decision-making following optogenetic inhibition of this region during discrete trial epochs: on separate sessions during the presentation of the informative cue that fully predicts reward (S+) or during the intertrial interval (ITI). As a positive control for the effectiveness of optogenetic inhibition of ACC, we evaluated animals on effort-based choices involving high versus low valued rewards, where its role has been documented extensively (Floresco and Ghods-Sharifi, 2007; Hauber and Sommer, 2009; Bailey et al., 2016; Mashhoori et al., 2018; Porter et al., 2019; Hart et al., 2020).

## Materials and Methods

### Animals

Twenty-four Long–Evans rats (*Rattus norvegicus*; *N* = 14 females) were acquired from Envigo (Bioanalytical Systems). Two animals were not included in the analyses due to unilateral expression of the virus, one due to wrong placement of the optic fiber and one on losing the headcap during the experiment (total *N* = 20, 10 females). A subset of animals (*n* = 11) was also tested on a progressive ratio (PR) task. Subjects were aged between postnatal day (P) 90 and 120 at the start of the experiment. Subjects were paired-housed before and separated after surgeries and housed in transparent plastic tubs with wood shaving bedding in a vivarium maintained on a reverse 12 h light cycle. Experiments were conducted at a minimum of 6 d per week during the dark portion of the light cycle. A progressive food restriction schedule was imposed prior to the beginning of the experiment to maintain rats at 85% of their initial free-feeding weights. Water was provided *ad libitum* in the homecage. The procedures used in this experiment were conducted under approval and following the guidelines established by the Chancellor's Animal Research Committee at UCLA.

### Viral constructs

An adeno-associated virus AAV9 driving an inhibitory opsin archaerhodopsin T under the CaMKIIa promoter was expressed in neurons in ACC (*n* = 15, AAV9-CaMKIIa-ArchT)-Green, packaged by Addgene, Addgene, viral prep #99039). A virus lacking the Arch-T gene and instead containing the fluorescent tag eYFP (*n* = 5, AAV9-CaMKIIa-EYFP, packaged by Addgene) was infused into ACC as a null virus control. All animals underwent identical surgical procedures, and all behavior was conducted in a counterbalanced within-subject design with each animal serving as its own control between inactivation sessions. This allowed us to account for nonspecific effects of surgery, exposure to AAV9, and nonspecific effects of laser stimulation.

### Behavioral apparatus

Behavioral testing was conducted in Med Associates operant conditioning chambers. Each chamber contained two retractable levers inserted to the left and right of a lower food delivery port in the front wall. A photobeam entry detector was positioned at the entry to the food port to detect head entries. A pellet dispenser connected to the food port delivered a single 45 mg sucrose pellet (Bio-Serv). Tone, clicker, and white noise generator were attached to individual speakers on the wall opposite the lever and magazine. A 3 watt, 24 volt houselight on the top of the back wall provided illumination. For optogenetic manipulations, the chambers were outfitted with an Intensity Division Fiberoptic Rotary Joint (Doric Lenses) connecting the output fiber optic patch cords to a 532 nm Green laser (Shanghai Laser & Optics Centur) positioned outside of the chamber.

### Surgical procedures

After completing the training phase, rats were anesthetized with isoflurane for bilateral infusion of ACC with either an inhibitory opsin archaerhodopsin T (AAV9-CaMKII-ArchT)-Green (Addgene, viral prep #99039) or eYFP (AAV9-CamKII-EYFP; Addgene). Craniotomies were created for the injection sites but also four extra holes were used to screw anchors. After the four screws were fixed in the skull, animals were infused with two bilateral sites of injections in ACC (0.3 µl at 0.1 µl/min AP: +3.7, ML: ±0.8, DV: −2.4 for a total volume of 0.3 µl per side). After infusion, the blunt needle was left in place for 10 min to allow diffusion. Using the same craniotomies, a stainless-steel fiber optic cannula ferrule (size: 1.25 mm, 200 μm Core, NA: 0.37, length: 2.5 mm; Thorlabs) was inserted in each side (AP: +3.7, ML: ±1.0, DV: −2.1, inserted in a 15° angle). All measurements were taken from the bregma. Optic fibers were secured in place with cyanoacrylate, bone cement (C&B Metabond, Parkell) and dental acrylic (Patterson Dental). Animals were provided with postoperative carprofen (5 mg/kg, s.c.; Zoetis) daily for 5 d.

### Behavioral procedure

#### Pretraining

Before training, 10 sucrose pellets (0.5 g) were provided in the homecage to accommodate rats to the food rewards. On the first day, rats were trained to eat pellets from the pellet tray by delivering 1 pellet every 20 ± 15 s in the chamber for a total of 40 pellets. On Days 2 and 3, rats were trained to lever press the left and right lever (lever presented in alternate order) in an autoshaping procedure. Reinforcements were delivered following each lever press. After pretraining, animals were either trained on the information choice task or the effort-based choice task, in counterbalanced order.

#### Information choice training

The training stage of the task was composed of two types of trials: choice and forced trials. In choice trials, rats were required to choose between two levers available simultaneously. A choice trial started with the simultaneous insertion of both levers and the houselight turning on. A choice was made by pressing a lever one time. After the choice was made, if the animal chose the Info option, both levers retracted and the houselight turned off. If the lever associated with the Info option was pressed, 20% of the time an auditory cue (S+) was presented for 30 s always ending with food; the other 80% of the time another auditory cue (S−) was on for 30 s never ending with food. If the rat chose the No-Info option, a third auditory cue (S3) was presented for 30 s and ended with food on half of the trials. On forced trials, only one lever was presented at a time, following the same contingencies described above. All trials were separated by a 10 s intertrial interval (ITI) during which all stimuli were off. A single session constituted 54 trials, 18 choice trials, and 36 forced trials. The assignment of sounds to S+, S−, or S3 and the lever side for each option was counterbalanced across animals. After 10 sessions, rats were connected to the patch cords without any stimulation to habituate them to move around and perform the task with the optical tether (200 µm, 0.22 NA, Doric Lenses) for another 6 sessions, with no light delivery. Thus, training was a total of 16 sessions.

#### Information choice test

**Figure 1.**
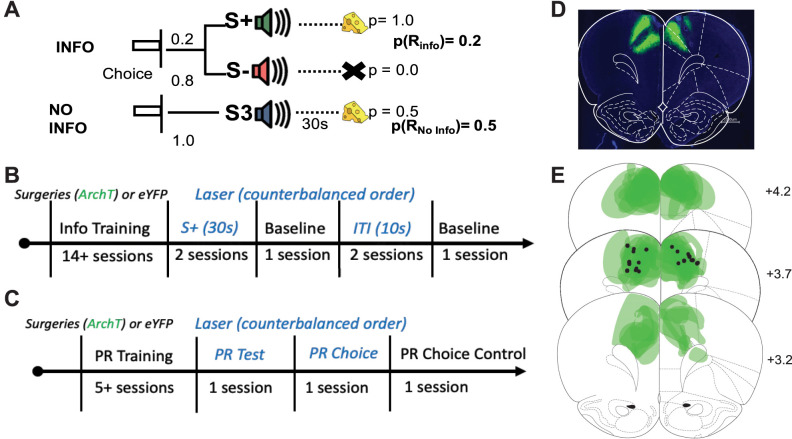
Information choice task, effort-based choice task, timelines, and histology. ***A***, Info choice task. Rats choose between two levers (left or right). If the rat chooses the “Info” option, on 20% of the trials, tone S+ plays for 30 s terminating in the delivery of one sugar pellet; the other 80% of trials tone S− plays, terminating in no food. If the rat chooses the “No-Info” option, a third tone S3 plays for 30 s, terminating in one sugar pellet on 50% of the trials. A session consisted on 54 trials evenly split between choice, forced-info, and forced no-info trials. Only one lever was available on forced trials. ***B***, Timeline Info task. Rats underwent surgery for virus and fiber implant prior to any training. Conditions with optogenetic inhibition were during the S+ and ITI, counterbalanced and alternated with a no-laser session (baseline) between conditions. During the S+ condition, ACC inhibition occurred in choice and forced-info trials, during the duration of the cue S+ (30 s). During the ITI condition, ACC inhibition occurred on 10% of the 10 s ITI epochs. ***C***, Timeline PR task. Rats underwent surgery for virus and fiber implant prior to any training. During PR Test and PR Choice, ACC inhibition occurred during sucrose pellet retrieval. PR Test was just as the training session. In PR Choice and PR Choice Control, animals were presented with a ramekin of 20 g of chow. The order of testing was counterbalanced across animals. A subset of animals experienced both tasks, in counterbalanced order. ***D***, Photomicrograph. Representative placement of inhibitory opsin ArchT (green) and fiber placement at anterior-posterior (AP) level +3.7 for ACC relative to Bregma. ***E***, Reconstructions. Reconstructed placements of inhibitory opsin ArchT virus (green) and optic fiber placement (black) across all animals, for AP level +4.2, +3.7 and +3.2 relative to the bregma, targeting ACC.

During two consecutive sessions, rats were tested on the task as described above but this time receiving optogenetic inhibition during either the 30 s S+ presentation or on 10% of ITI periods (to equate to the duration of other trial epochs and minimize potential damage due to prolong photoinhibition). Green light was delivered into ACC, which inhibited pyramidal neurons in this region during these trial epochs in rats expressing ArchT and not eYFP controls. During each presentation, a green light (532 nm; 10 mW) was delivered via a laser (532 nm; Shanghai Laser and Optics Century) connected through a ceramic mating sleeve (2.5 mm; Precision Fiber Products) to the ferrule implanted in the rat. Optogenetic parameters were chosen considering previous work on the duration of continuous inhibition in ArchT-expressing neurons (up to 1–15 min; Huff et al., 2013; El-Gaby et al., 2016) compared with our 30 s inhibition, and considering that cell body inhibition as we conduct here is more selective, limiting off-target effects compared with terminal inhibition (Lafferty and Britt, 2020). Light leakage from laser output was prevented using 5-cm-long black shrinktube shielding over the connected patch cord and cannula ferrules. Between each condition, animals received a baseline session, in which no inhibition was administered but animals were connected to the optical tether as before. The order of inhibition during the three different epochs was counterbalanced (Fig. 1*B*).

#### Progressive ratio training (for effort-based choice)

Animals were trained on a progressive ratio (PR) schedule where the required number of presses for each pellet increased according to the formula:

$$n_{i}=5e^{\left(i/5\right)}-5.$$

As described in Hart et al. (2020), the effort requirement increased for the rat on earning five pellets where $n_{i}\;$ is equal to the number of presses required on the *i*th ratio, rounded to the nearest whole number after five successive schedule completions. Each session started with the insertion of the left lever. The completion of a ratio requirement was marked by a light, which remained on until the animal collected the pellet from the food tray. A session lasted 90 min. The training lasted a minimum of five sessions and continued until stability was reached, defined as the average number of lever presses in a session not varying by >15% across the last two sessions.

#### PR Test

Animals experienced two test sessions in which ACC was inhibited, using the same parameters described in the Info choice test. In one test, animals underwent a PR session similar to the training phase, during which inhibition occurred during pellet retrieval, and where sucrose pellets were the only option. Our previous imaging of single cells in rat ACC revealed a phasic increase in activity during reward collection of the preferred reward (Hart et al., 2020); thus, we predicted that inhibiting ACC during this trial epoch would be particularly effective in changing effort-based decision-making. In another test session, animals received a PR Choice session where the task occurred as described, but rats also had free access to 20 g of chow in a ceramic ramekin positioned on the right side of the food port (opposite the lever location, along the same wall as the pellet tray). For both tests, the onset of the inhibition occurred at pellet delivery and lasted for 2 s or until the animal collected the pellet or pressed the lever (whichever occurred first). Animals also underwent a PR Choice control session identical to PR Choice but without laser inhibition. The order of test sessions was counterbalanced (Fig. 1*C*).

### Histology

At the conclusion of the experiment, rats were killed with an overdose of sodium pentobarbital (Euthasol, 0.8 ml, i.p.; VetOne) and transcardially perfused with phosphate-buffered saline (PBS) followed by 10% buffered formalin acetate. Brains were extracted and postfixed in this solution for 24 h followed by 30% sucrose immersion. Tissue was sectioned in 40-µM-thick slices and coverslipped with DAPI mounting medium (ProLong Gold, Invitrogen) visualized using a BZ-X710 microscope (Keyence), and analyzed with BZ-X Viewer software. A representative image of the viral expression and implant tracks in the Arch-T group is shown in Figure 1*D*. Reconstructions of the viral expression across all Arch-T expressing animals are shown in green with black dots indicating the location of the bottom of the optic fiber implants across animals (Fig. 1*E*).

### Data analysis

All data processing and visualization were conducted using R Studio and GraphPad Prism 10 (GraphPad Software). All data were subsequently analyzed via custom-written code in MATLAB (MathWorks). For the Info choice task, we analyzed three main variables: (1) preference for the Info option (Info choice divided by the total number of choices), (2) latency to choose (the time from the onset of the trial until lever press), and (3) port entry rate (the number of entries into the food port) during the 30 s cue duration. For all analyses, we used mixed-effects general linear models (GLMs; *fitglme* function; Statistics and Machine Learning Toolbox) to more rigorously handle data interdependencies (de Melo et al., 2022; Yu et al., 2022) such as cue exposure that depended on individual rat's choice. Preference data were analyzed first in omnibus GLM that included all factors (sex, trial epoch, laser on/off) and all groups (virus: Arch-T vs control). Sex was included as a moderator in training and a covariate at test. Follow-up analyses were further pursued pending significant interactions in the full model with planned comparisons: S+, S−, S3 and PR, PR Choice. Coding of variables for GLMs was as follows: 0 = females and 1 = males; 0 = control and 1 = ArchT; 0 = ITI, 1 = S+; 0 = laser off (no laser), 1 = laser on (laser on in the current trial). Individual rat was entered as a random factor. Latency analysis included the described variables in addition to the within-subject variable: trial-type (forced-info, forced-no info and choice). Port entry rate analysis included cue as a crossed, within-subject variable (S+, S−, and S3).

For the PR task, we analyzed three main measures: (1) mean lever presses in a session; (2) breakpoint (BR), that is, highest incomplete PR ratio; and (3) latency to collect reward. As in the information task, we used GLM planned comparisons to probe significant interactions, when observed. To assess correlations between information preference and performance on the effort task, we conducted Spearman rank analyses. For this we included only animals that had been involved in both tasks on only laser-inhibited trials and included both types of effort contexts (PR and PR Choice). All code and data can be found in the following repositories: https://github.com/izquierdolab and https://gin.g-node.org/aizquie.

## Results

### Information choice training: rats acquire a stable preference phenotype, and respond faster in choice trials

#### Preference

We found that rats exhibit a stable preference phenotype that does not change by the end of training. Training data were analyzed using preference across sessions. A GLM was performed for the outcome variable, information (Info) preference, using session as within-subject factor, sex as a between-subject factor, and individual rat as a random factor using the formula: *γ* ∼ [1 + sex * session + (1 + session| rat)]. We found no significant predictors or interactions with session (GLM: *β*_session _= 0.005, *t*_(290)_ = 0.65, *p *= 0.51), sex (GLM: *β*_sex _= 0.025, *t*_(290)_ = 0.40, *p *= 0.68), or session * sex (GLM: *β*_session * sex _=0.0002, *t*_(290)_ = 0.02, *p *= 0.98).

**Figure 2.**
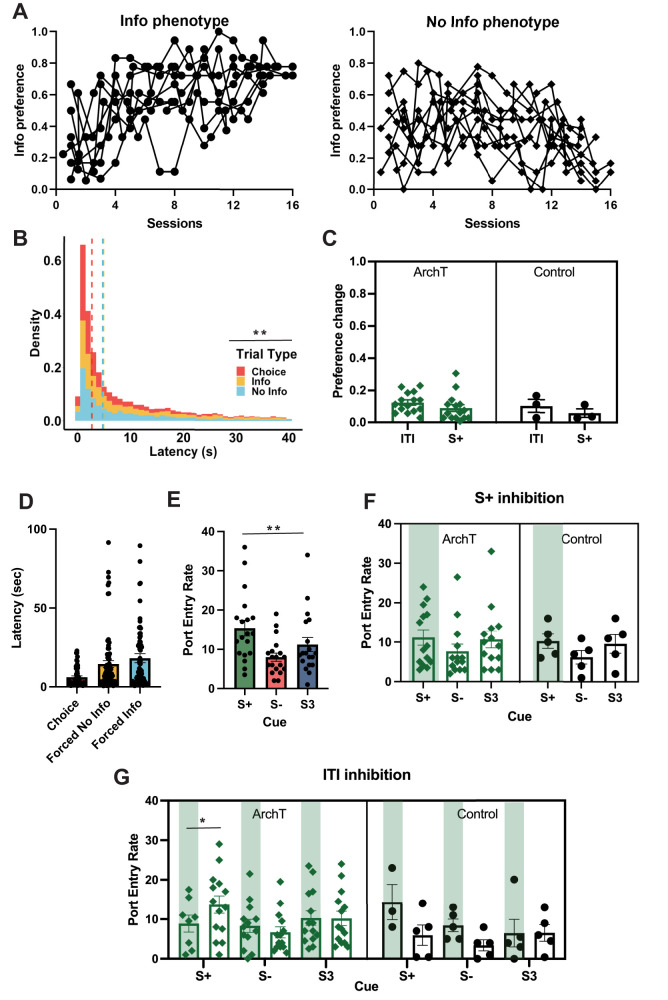
Optogenetic inhibition of ACC prospectively reduces reward expectation during the Info (S+) cue. ***A***, Establishment of phenotype in animals during training. Each line represents an individual rat. ***B***, Density of the total number of observations for latency to choose by trial type collapsed across conditions during training. Dashed lines refer to median latency. Median latency to choose in choice trials (red line) was faster than in forced trials (gray line). ***C***, Mean absolute preference change from baseline (no laser) during inhibition of S+ cue or ITI presentation, with individual rats represented as scatter. ***D***, Median latency to choose and during forced No Info and forced Info trials at test. ***E***, Median port entries during the 30 s cue presentations (S−, S+, and S3) for all animals. Individual circles correspond to median responses for each animal. ***F***, S+ inhibition: Median port entries during each cue presentation on laser on (green vertical stripes) and off (no stripes) for ArchT (left, green) and control (right, black) animals, with individual rats represented as scatter. ***G***, ITI inhibition: Median port entries during each cue presentation following laser on (green vertical stripes) and off (no stripes) for ArchT (left, green) and control (right, black) animals, with individual rats represented as scatter. A decrement in port entries during S+ was observed following inhibition in the ArchT group, but not the Control group. There was a general increase in port entry rate in laser on trials in the Control group, but it was not specific to a cue. Error bars denote ± SEM. **p < 0.01, *p < 0.05. ArchT *n* = 11, Control *n* = 5.

The absence of a significant effect of session may be due to the high variability of choices in the development of a preference. To address this, we classified animals based on the phenotype exhibited during the last three sessions of training: Info preferring (preference above 0.6), No Information preferring (No Info; preference below 0.4), and Indifferent (between 0.4 and 0.6). Using phenotype as a fixed factor moderator in the formula *γ* ∼ [1 + sex * phenotype * session + (1 + session| rat)], we found a significant effect of session (GLM: *β*_session _= 0.033, *t*_(286)_ = 2.71, *p *= 0.007) and a significant session * phenotype interaction (GLM: *β*_session * phenotype _= −0.023, *t*_(286)_ = −2.68, *p *= 0.007). This interaction indicates that preference changed differentially across sessions of training. Animals began their preference around the indifference point 0.5 but then diverged, with a group of animals remaining at the indifference point and the other two groups developing a strong preference toward/against the Info option (Fig. 2*A*). When selecting the last three sessions of training to check for stability, use of the same formula led to no significant predictors or interactions, confirming animals’ preference was stable by the end of training.

#### Latency to choose

We found that rats are faster to make choices than to complete forced trials, corroborating prior research (González et al., 2023, 2024). We conducted a GLM on median latencies using trial type (forced Info, forced NoInfo, and Choice) as within-subject factors, sex as between-subject factor, and individual data as a random factor, formula: *γ* ∼ [1 + trial type * sex + (1 + trial type| rat)]. The results yielded a significant main predictor of trial type (GLM: *β*_trial type _= 5.19, *t*_(68)_ = 3.79, *p *< 0.001), but no effect of sex (GLM: *β*_sex _= 1.43, *t*_(68)_ = 0.52, *p *= 0.60), or interaction (GLM: *β*_trial type * sex _= −0.167, *t*_(68)_ = −0.60, *p *= 0.55). Indeed, rats are faster during choice trials (Median_Choice _= 4.4 s, SEM ± 0.81) than forced trials (Median_Forced Info _= 11.4 s, SEM ± 2.46; Median_Forced NoInfo _= 7.6 s, SEM ± 2.64; Fig. 2*B*).

#### Port entry rate

Port entry rate is an indication of differential reward expectation for S+ versus S−. We analyzed the median port entry rate (entrance to the feeder port) for each 30 s cue duration for the last three sessions of training. A GLM was conducted for median port entries using sex as a between-subject factor, and individual rat crossed with cue (S+, S−, and S3) as a random factor, according to the following formula: *γ* ∼ [1 + sex * cue + (1|rat:cue)]. Cue was included in this way to account for the fact that rats experienced each cue a different number of times given the inherent contingencies of the task: S+ was presented on only 20% of all Info trials but it also depended on the rat's choices (for instance, a rat that chooses the No Info option exclusively would have an overrepresentation of the S3 cue and very few S+ trials). We found no effect of cue or sex on port entry rate (*p* > 0.64). We have previously reported the greatest responses for the S+ and lowest responses to S− (González et al., 2024). However, in that study, the cue duration was 60 s, affording a longer window for differences to emerge.

### Information choice test: optogenetic inhibition of ACC neurons decreases port entry rate during S+ cue but does not affect overall information preference or latencies

#### Preference

Info preference during test sessions was analyzed for both epochs. Mean values were used for S+ and ITI inhibition sessions. The GLM formula was *γ* ∼ [1 + virus * laser * epoch + sex + (1 + epoch + laser| rat)], with epoch (S+ or ITI inhibition) and laser entered as within-subject factors, and sex as a covariate. None of these factors or their interactions significantly predicted Info preference. Given that we observed high variability in the initial preference, any detectable changes in preference depended on each rat's baseline. Therefore, we calculated the absolute change in preference during each optogenetic inhibition session minus the preference during baseline, identical to the analysis in González et al. (2024). The same GLM formula was used but instead we used the absolute preference change as the outcome of interest. No significant predictors or interactions between factors was found. Similarly, these same factors did not predict phenotype. In summary, optogenetic inhibition of ACC did not affect overall preference or change in preference phenotype (Fig. 2*C*).

#### Latencies

Optogenetic inhibition of ACC could affect performance measures such as latencies, not just preference per se. To test this, we analyzed median latencies to choose during test sessions. Here we used the formula: *γ* ∼ [1 + virus * epoch * trial type * laser + sex + (1 + epoch + trial type + laser| rat)], where virus and sex were between-subject factors and epoch (S+, ITI, and Baseline), trial type (Choice, forced Info and forced No info), and laser (laser on, off) were within-subject factors, and individual rat was a random factor. The results revealed no significant predictors or interactions (Fig. 2*D*).

#### Port entry rate

Median reward port entries were analyzed for S+ and ITI epochs using the following GLM formula: *γ* ∼ [1 + virus * cue * laser + (1 + laser| rat:cue)] and separately for the baseline condition with laser and virus not included in the formula because laser was not yet introduced: *γ* ∼ [1 + cue + (1 | rat)]. The analysis of baseline sessions across all animals resulted in a significant effect of cue (GLM: *β*_cue _= −2.09, *t*_(55)_ = *p *= 0.005; Fig. 2*E*). The analysis of S+ inhibition (with cue left out of the model) resulted in no significant effects (*p *> 0.17) or interactions (*p *> 0.88; Fig. 2*E*). However, inhibition during the ITI epoch resulted in a significant effect of laser (GLM: *β*_laser _= 9.87, *t*_(97)_ = 2.66, *p *= 0.009), a virus * laser interaction (GLM: *β*_virus*laser _= −14.97, *t*_(97)_ = −3.45, *p *= 0.0008), and virus * cue * laser interaction (GLM: *β*_virus * cue * laser _= 5.21, *t*_(97)_ = 2.72, *p *= 0.007). Due to these interactions, we separately investigated the effect of inhibition in the ITI epoch by virus, where we used this GLM formula for predicting median port entry rate: *γ* ∼ [1 + cue * laser + (1 + laser| rat:cue)].

The analysis for the ArchT, but not controls, resulted in a laser * cue interaction (GLM: *β*_laser * cue _= 2.13, *t*_(73)_ = 2.15, *p *= 0.035), indicating the inhibition differentially affected responsivity to cues. Planned comparisons for each cue resulted in a significantly lower port entry rate during the subsequent S+ (GLM: *β*_laser _= −4.65, *t*_(20)_ = −2.33, *p *= 0.03), but not S− (*p *= 0.20) or S3 (*p *= 0.89; Fig. 2*F*). The analysis of the control virus group resulted in a significant effect of laser (GLM: *β*_laser _= 9.119, *t*_(24)_ = 2.48, *p *= 0.020), with animals checking the food port more (not less) following all “laser on” trials, irrespective of the cue presented. This effect was not explained by animals being distracted by residual light, given that the responses during cue presentation occurred after the laser was experienced and could instead reflect enhanced arousal and activity levels following laser stimulation in controls. In sum, ACC inhibition led to a prospective decrease in reward checking specifically during the S+, indicating that animals did not exhibit the appropriate expectation of a certain reward.

### Progressive ratio: ACC inhibition reduced effort and decreased breakpoint, without affecting latency to retrieve reward

#### Total lever presses

**Figure 3.**
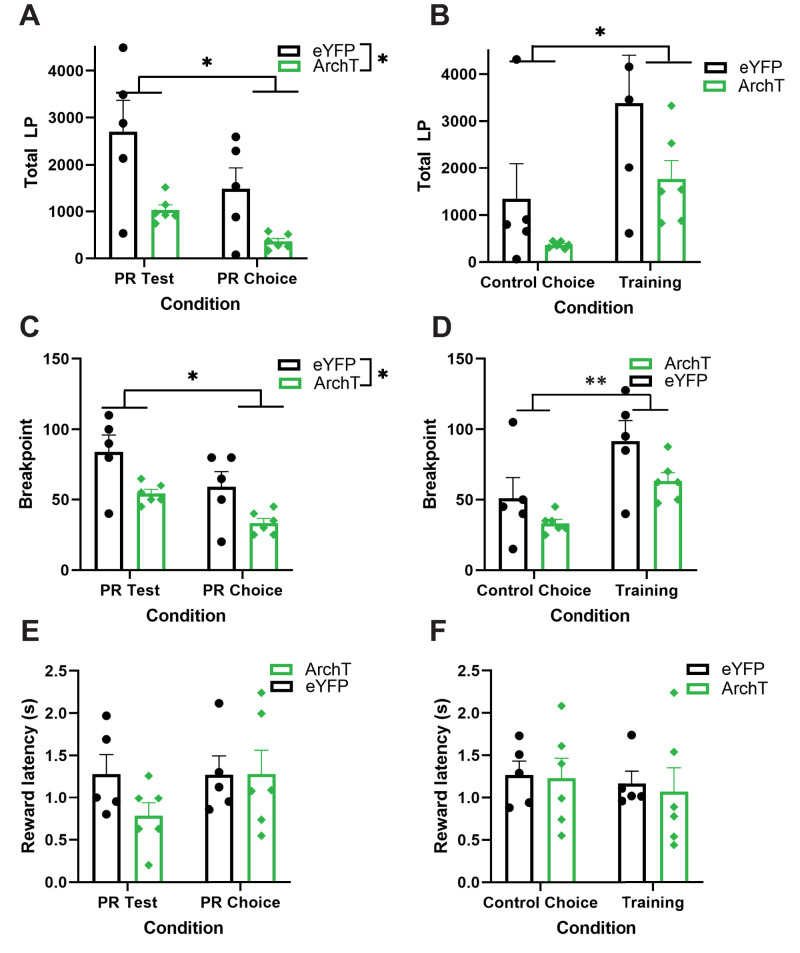
Optogenetic inhibition of ACC during PR and PR Choice decreases effort without affecting motivation. ***A***, Mean total lever presses during PR Test and PR choice for ArchT (green) and control (black) animals. Individual rats represented as scatter. ***B***, Mean total lever presses during control Choice (PR choice with laser off) and last session of training for ArchT (green) and control (black) animals. Individual rats are represented as scatter. Lever presses during Control choice test were lower than during training, with no difference between virus group. ***C***, Mean BP during PR Test and PR choice for ArchT (green) and control (black) animals. Responses were lower during PR choice than PR Test, but also lower for ArchT animals. ***D***, Mean BP during Control choice and Training (no inhibition) for ArchT (green) and control (black) animals. Individual rats are represented as scatter. A significant difference of test but not virus was found. ***E***, ***F***, Mean latency to collect reward during inhibited trials (PR Test, PR Choice) and during noninhibited trials (control choice and training) for ArchT (green) and control (black) animals, with individual dots corresponding to median latency for each rat. No difference in reward latencies were observed. *p < 0.05, ***p < 0.01. ArchT *n* = 6, Control *n* = 5.

We analyzed total lever presses during sessions with laser on during the reward collection period. This epoch was selected based on previous work from our lab implicating phasic increases in population activity in ACC for higher-valued rewards as essential for effort-based decision-making (Hart et al., 2020). In these analyses, virus was a between-subject factor, test (PR Test and PR Choice Test) was a within-subject factor, sex was a covariate, and individual rat was entered a random factor. The GLM formula was as follows: *γ* ∼ [1 + virus * test + sex + (1 + test| rat)]. We found a main effect of test (GLM: *β*_test _= −1,229.6, *t*_(17)_ = −4.02, *p *= 0.0008), an effect of virus (GLM: *β*_virus _= −2,191, *t*_(17)_ = −2.42, *p *= 0.027) but no effect of sex (*p *= 0.30) or interactions (*p *= 0.19), indicating that responses during the PR Test were higher than during PR Choice Test but also that overall responses were lower for the ArchT virus group compared with the control animals (Fig. 3*A*). We also compared performance during sessions with no laser (last session of training and PR Control Choice). For this, the GLM formula was identical to the previous analysis, and here we found a significant effect of test (GLM: *β*_test _= −678.4, *t*_(17)_ = −4.86, *p *= 0.0001), also resulting in a higher level of responding during PR training than PR Control Choice, but no effect of virus (*p *= 0.09), sex (*p *= 0.22), or interactions (*p *= 0.28; Fig. 3*B*). These results collectively show the expected effect that animals tend to work more vigorously when sucrose pellets (i.e., in PR training or PR Test, but not PR Choice or PR Control Choice) are the only option. More importantly, these data indicate that ACC inhibition reduced effort and that this decrement in effort was not due to a general reduction in motivation per se, given that rats still lever press more when sucrose was the only reward (i.e., the decrement was proportional to their initial performance in the given test). If ACC inhibition was affecting motivation, we would expect a similar level decrement in PR Test and PR Choice.

#### Breakpoint

The last incomplete PR step was noted as the breakpoint. We analyzed breakpoint for the two tests with inhibition (PR Test and PR Choice), with an identical GLM formula as for lever presses: *γ* ∼ [1 + virus * test + sex + (1 + test| rat)]. This analysis also yielded a main effect of virus (GLM: *β*_virus _= −33.30, *t*_(17)_ = −2.32, *p *= 0.033), and test (GLM: *β*_test _= −25, *t*_(17)_ = −4.92, *p *= 0.0001), but no effect of sex (*p *= 0.46) or interactions (*p *= 0.55; Fig. 3*C*). For the test sessions without laser (PR Training and PR Control Choice), we used the same formula as above. Here we found an effect of test (GLM: *β*_test _= −13.5, *t*_(17)_ = −7.04, *p *= 2.00 × 10^−6^), but no other effects (*p *> 0.05) or interactions (*p *= 0.18; Fig. 3*D*). Taken together, this confirms that the decrement in effort following ACC inhibition depended on the availability of another source of reward.

#### Latencies

We analyzed median latencies to collect the reward. As before, we analyzed the two tests where inhibition occurred using the following GLM formula: *γ* ∼ [1 + virus *  test + sex + (1 + test| rat)], where *γ* was the median latency to collect reward, virus was a between-subject factor, test was a within-subject factor, sex was entered as covariate, and individual rat was entered as a random factor. Comparing PR Test and PR Choice Test, we found a significant effect of sex (GLM: *β*_sex _= −0.40, *t*_(17)_ = −2.45, *p *= 0.026), where females were faster at collecting reward than males, yet no other significant predictors (*p *> 0.089) or interactions (*p *= 0.22) were observed (Fig. 3*E*). Similarly, for PR training and PR Control Choice, no significant effects (*p *> 0.13) or interactions were found (*p *= 0.74; Fig. 3*F*). Collectively, these results suggest that the decrement in effort cannot be explained by a general reduction in motivation to the rewards, where we would expect an increase in latencies to collect pellets.

### Correlations between info preference and effort

Spearman rank correlation coefficients were computed to assess the relationship between the main measure of information preference and all other main dependent measures in the PR task (both PR and PR choice). Expectedly, we found significant correlations within the effort-based task itself, with positive correlations between median lever presses and total lever presses (*r* = 0.899, *p *= 0.028) and total lever presses with breakpoint (*r* = 0.986, *p *= 0.006). Interestingly, the only significant association with information preference was median reward latency in the PR task (*r* = −0.899, *p *= 0.028), which was a negative association: animals that had a strong information preference were quicker to collect reward on the PR task (Table 1).

**Table 1.** Info preference and effort-based choice (Spearman rank correlation coefficients)

	InfoPref	Median LP	Total LP	Reward latency	Breakpoint
InfoPref	1.0				
Median LP	0.388	1.0			
Total LP	0.429	**0.899** *	1.0		
Reward latency	**−0.899** *	−0.662	−0.580	1.0	
Breakpoint	0.377	**0.956** *	**0.986** **	−0.588	1.0

* *p* < 0.05.

** *p* < 0.01.

Bold text is significantly correlated.

## Discussion

We investigated the role of ACC in information and effort choices. Previous research has highlighted the involvement of ACC in decision-making processes, particularly in evaluating effort costs (Hosokawa et al., 2013; Bailey et al., 2016; Hart et al., 2017, 2020; Fatahi et al., 2020; Cai and Padoa-Schioppa, 2021), so we used this behavior as a positive control for the efficacy of our optogenetic manipulation in Info choices. In previous studies, we and others have found ACC to be important in maintaining a representation of the value of both options in a 2-alternative choice paradigm (Hart et al., 2017, 2020; Mashhoori et al., 2018). The present results from both tasks suggest that ACC is required to maintain the appropriate reward expectation, either for the higher-valued reward of sucrose (in the effort task) or for the more certain reward (in the Info task). Interestingly, inhibiting ACC neurons decreases this stay-on-goal behavior without changing motivational value per se because reward latencies were unaffected by inhibition. Based on the results of this experiment, we suggest that this may be the mechanism by which stable preference is maintained in Info choices (González et al., 2024). The ITI would be the crucial epoch in the task where previous trial experience gets consolidated to update the course of future action. Indeed, a preference for information on the Info task was correlated with shorter reward latencies in the effort task, indicating these two measures are related via reward expectation.

Despite the effect of inhibition on reward expectation at the trial level, we were surprised to find no change in global Info preference. However, in order to equate overall inhibition time between S+ and ITI epochs, we only inhibited ACC on 10% of the 10 s ITIs. Had we inhibited ACC neurons on more of these ITIs, we may have observed an effect on overall preference. In keeping with the literature, we did observe distinct behavioral phenotypes for information choices: most rats either exhibited a strong preference for the Info option or preferred the No-info option. Almost no animal remained indifferent to the choice options. This divergence suggests that information may be valuable, but when there is a cost, such as via a potential loss of reward opportunities, there are individual differences in that cost sensitivity. Future experiments could modulate or titrate this cost sensitivity as a continuous variable.

Our present findings may intersect with the literature on sign- versus goal-tracking as there are relevant discoveries worth noting in that vein. For example, (1) both uncertainty reduction and conditioned reinforcement influence information choice (Ajuwon et al., 2023); (2) strong stimulus-reward predictive utility promotes information preference (Chow et al., 2017); and (3) individual differences in sign- versus goal-tracking do not fully predict information preference in rats (Lopez et al., 2018). We expected that ACC would be robustly involved in this task due more to its involvement in uncertainty monitoring rather than any role in conditioned reinforcement. Unlike measures of decision confidence assessed by temporal wagering for reward (Lak et al., 2014; Stolyarova et al., 2019), in this task the rat makes a choice prior to cue presentation and the auditory cue remains on from the moment the rat has selected the option until the outcome is revealed (i.e., reward or no reward). As a result, animals are not relying on memory and do not need to keep the cue “in-mind.” Therefore, because inhibition of ACC neurons reduces subsequent checking of the reward port when there is certain to be a reward (S+), this is evidence neurons in this region support reward expectancy rather than uncertainty monitoring in this task. Better goal maintenance could facilitate exploration, grooming, or resting, etc., ultimately supporting more behavioral flexibility under certainty.

We and others (Cox et al., 2023) have previously proposed that ACC is important in accessing the relative value of each option, which as mentioned above requires a representation of both outcomes. In support of this, we find that ACC inhibition decreased reward expectation for a certain reward by affecting goal maintenance. Recent work by Vázquez et al. (2024) proposed that ACC is important for attention-related processes, contributing to the ability to “stay on task” (Vázquez et al., 2024). Recent work by Regalado et al. (2024) is also consistent with our findings. This group investigated the role of neural activity in ACC when mice self-initiate trials to learn cue–reward contingencies. Authors found that neural activity in orbitofrontal cortex-to-ACC projections increased progressively with consecutive unrewarded cues and peaked at a reward-associated cue, keeping the animal “on track” in the task. Most relevant to the present study is that ACC maintains the relevant goal information to allow for such goal-directed behavior.

On the effort choice task, ACC inhibition had no impact on the speed of reward collection but did reduce effort. This aligns with the extensive literature implicating ACC in effort-related decision-making, supporting the idea that ACC activity is integral to sustaining high-effort behavior for high-value rewards (Hosking et al., 2014; Bailey et al., 2016; Azab and Hayden, 2017; Fatahi et al., 2020; Cai and Padoa-Schioppa, 2021). Consistent with what was observed in the Info choice task, the lack of changes in latencies to collect reward suggest that the motivational value of the reinforcer remained intact. Furthermore, the decrement in effort during ACC inhibition was proportional to the type of test, that is, the effort was lower when an alternative source of reward was introduced (PR Choice) compared with when only sugar pellets were available (PR Test). If ACC inhibition was involved in altering motivation, a similar level of effort would have been observed if either one or two sources of reward were present. Instead, the pattern of responses was preserved and did not affect speed of retrieval, suggesting a disruption in maintaining the goal of the better option (i.e., sucrose). We have previously shown sucrose is indeed the better option when the choice is between chow and sucrose pellets (Hart et al., 2017, 2020).

In summary, while ACC inhibition did not directly alter Info preference or reward latencies, it significantly affected reward expectation during an informative (S+) cue and attenuated effort-based choice. These results underscore a nuanced role for ACC in information-seeking behavior and effort-based choice via a common stay-on-goal mechanism. Future studies should explore more precise temporal and spatial dynamics of ACC activity to further elucidate its role in these complex behaviors.

## Synthesis

Reviewing Editor: Mark Laubach, American University Decisions are customarily a result of the Reviewing Editor and the peer reviewers coming together and discussing their recommendations until a consensus is reached. When revisions are invited, a fact-based synthesis statement explaining their decision and outlining what is needed to prepare a revision will be listed below. The following reviewer(s) agreed to reveal their identity: Matthew Roesch, Rachel Anderson.

Thank you for sending your work to eNeuro!

Your manuscript was reviewed by two experts and their reviews are given below in full. Please revise your paper to address all points that were raised. In addition, please revise with regards to three comments (from me) below.

1. Please consider including a graphical summary of the study, such as a figure highlighting the optogenetic effects and the first panel of Figure 1. This visual representation would be helpful for promoting the study on social media.

2. The manuscript does not explicitly mention code availability. While the study was not computational, the journal encourages sharing code and data from accepted articles. Please consider providing an example of your data analysis in your revised submission as Extended Data or post your code to a repository.

3. There seems to be a discrepancy in the methods section regarding the software used for data processing and analysis. It states, "All data processing was conducted using R Studio. All analyses were performed via custom-written code in MATLAB." As a user of both platforms, I'm unclear about how MATLAB was integrated into the RStudio workflow.

Please clarify whether:

- MATLAB code was executed within RStudio using the R.matlab package.

- Separate analyses were performed in R and MATLAB.

- A different approach was used.

A clear description of the data analysis pipeline and sharing code and data would enhance the reproducibility of the study.

Summary of main points from the two reviewers:

Reviewer 1:

- Rat behavior: Elaborate on why rats prefer the informative option and discuss the role of sign/goal tracking.

- ACC inhibition: Provide further rationale for connecting response rates to reward expectation and discuss why ACC inhibition during the ITI specifically impacts performance.

- Optogenetic parameters: Address concerns about the duration of ACC inhibition (30s).

- Correlation between tasks: Explore potential correlations between performance in the information task and the progressive ratio task.

- ACC and uncertainty: Discuss the unexpected lack of ACC involvement in uncertain decisions.

Reviewer 2:

- Methods clarity: Clarify the training schedule description.

- Data visualization:

- Show the latency data for the information choice test.

- Clarify the lack of significant difference in port entry rates between S+ and S- cues.

- Investigate the increased port entry rates in the control group during laser-on trials.

- Ensure Figure 3 is referenced in the text.

- Figure details: Improve figure clarity with details like individual rat identification, legend explanations, and significance markings.

- Result interpretation: Clarify the interpretation of the progressive ratio results, particularly the effect of ACC inhibition on lever pressing.

Full reviews:

Reviewer 1

I enjoyed reading this article and thought the task was interesting and finding in ACC significant. I did find the result surprising as did the authors. The task was to look a decisions that involve choosing between two options, one that leads to cues that predict exactly if reward is coming or not (i.e., informative cue), and the other tells the rat that reward will occur at 50% of the time. Overall, it would be better to go with the 50% reward, as opposed to the informative option because the S+ only occurs 20% of the time. Interestingly, rats come to choose the informative cue, suggesting that rats value information. The authors use optogenetics to test if ACC is important for this decision, which given what we know about ACC you would guess that it would! Surprisingly, the authors report that the choice between info and non-info options really isn't affected and they only see changes in response rates to the S+ cues that were presented after selection of the informational cue. It is also surprising that they really only see effects when ACC is inhibited during the ITI. They interpret these results as ACC being important for maintaining reward expectations and goal maintenance during expected reward (S+) in the service of maintaining info/ certainty seeking phenotypes. Seems like a little bit of a stretch but not unreasonable. They go on to show the ACC is important for effort, consistent with previous reports, which is nice to see. I do have some comments that could make the paper better in my opinion.

-I was wondering if it could be argued that the rats pick the info option more because they then know what is coming, which then frees them up to do other things (e.g., scratch, relax, look around)? I guess that is still the value of certainty and information, but I'd be curious to hear the author's thoughts about why they seek this option.

-It would be nice to have more rationale why response rates during cues tell us about "reward expectation", "goal maintenance" and "persistence toward rewards signaled by certainty". Perhaps plotting port entries over time leading up to the reward might be informative. It might be the case that after opto, rats were more time sensitive and waited to the end of the cue period to enter the port to collect reward. If that were the case, then ACC inhibition might not be impacting reward expectation.

-I was curious if sign/goal tracking came into play here. It seems like the rats might be sign-tracking in the sense they want the cue that predicts reward the best (i.e., S+; incentive salience). This would increase over sessions as the authors show. Also, ACC inhibition might make rats more sign tracky, thus they look at the S+ more instead of going to the food cup to receive reward.

-As the authors point out, it is surprising that effects were only seen during the ITI. More discussion of the possible mechanisms would be nice. Why does turning off ACC during the ITI impact performance?

-Are the authors concerned about the length of time the ACC was inhibited? 30s seems like a long time?

-Were the rats that performed the info task the same that performed PR? If so, it would be interesting to look at correlations between the two tasks to better understand what is going on in the info task during control and inhibition sessions. For example, is there a correlation between breakpoint (PR task) and response rates during S+ (info task)?

-I thought ACC would be more important for uncertain decisions not certain ones (e.g., conflict, decision confidence). I was surprised there was little discussion of this.

Reviewer 2

The paper "A common stay-on-goal mechanism in anterior cingulate cortex for information and effort choices" investigates the role of the ACC in persisting in reward seeking to a known outcome. The investigators use two tasks to assess this. In the first, rats are given two options: to gain information regarding the reward outcome or to have no information regarding reward outcome. Rat preferred getting information to no information. When the ACC was inhibited during the ITI interval, reward seeking was impaired after the S+ cue. ACC inhibition during the S+ cue itself did not alter reward seeking behavior. However, once the reward was available the latency to get the reward itself did not change providing evidence that the motivation for the reward didn't change.

In the second task, rats underwent a progressive ratio to reward task. During the testing phase, the ACC was inhibited either during reward retrieval. On some trials, only the reward was available. In others, the animals had access to rat chow. Rats pressed the lever more when there wasn't an option of food in all conditions but pressed the lever even less after ACC inhibition. Reward latencies again were unchanged, indicating that motivation remained intact.

Overall: I believe that the tasks outlined do a good job of investigating the question and the results are analyzed and interpreted correctly. There are, what appear to be, items overlooked that most certainly need to be addressed prior to publication and they are listed below.

Moderate/Minor Concerns

Materials and Methods:

Information choice test:

- Lines 140/141: The last sentence "Training continued for 14 sessions" feels misleading/confusing because it makes it seem as if another 14 sessions occurs following the 10 initial session and then the subsequent 4 sessions where animals were connected to the patch cords.

Results:

Information choice test: Latencies:

- These results are not significant, but an important part of the argument and are not graphed. The latencies for PR are graphed however. To make this uniform these should be graphed as well.

Port entry rate:

-Line 254: I find it odd that there isn't a significant difference in port entry rates between S+ and S- cues. I understand that the number of trials an individual animal experiences will be impacted by their choice and the nature of the task, but the fact that animals make equivalent port entries to both cues to me indicates that there isn't established learning of the various cues? That animals "expect" a reward for S- at the same level that they expect a reward for S+. Maybe I am overthinking this but I think this could be clarified for the more non-initated.

-Line 295/296: I also am curious as to why laser on in the control group would lead to increased port entry rates regardless of cue. Again, to me this indicates something about what an animal has learned about the task and puts into question the interpretation of the results. I am not considering this a major concern but I do think there is something here that should be looked into/investigated further. Additionally, these significant differences are not marked on the corresponding figure and should be (2F)

Progressive Ratio

-Figure 3 not referenced at all in text and should be

Figures

-2A: I am assuming each line represents an individual rat. This could be clarified or indicated with different colors per rat for easier reading.

-2B: Figure text should mention what the dashed lines represent (red for choice, grey for no choice latencies)

-2F: As indicated previously, significance should be indicated on the figure to show that port entry rates increased during laser on in control rats.

-3A: Line in the text says "There was an overall decrement in response in PR Choice compared with PR test with this decrease significantly more pronounced in ArchT animals". I find this misleading. In the body of the paper, this explained as there is an effect of test and virus but no interaction which to me indicates that there is less lever pressing in PR choice for both groups and less lever pressing for ArchT animals but not a larger decrease of lever pressing in PR choice of ArchT animals. This should be clarified.

-3D: significance of test should be indicated on figure
